# Testicular pathological alterations associated with SARS-CoV-2 infection

**DOI:** 10.3389/frph.2023.1229622

**Published:** 2023-06-29

**Authors:** Judy Ly, Rafael K. Campos, E. Eldridge Hager-Soto, Vidyleison N. Camargos, Shannan L. Rossi

**Affiliations:** ^1^ Department of Pathology, University of Texas Medical Branch, Galveston, TX, United States; ^2^ Department of Microbiology and Immunology, University of Texas Medical Branch, Galveston, TX, United States

**Keywords:** SARS-CoV-2, testes, male reproductive tract, testicular disease, COVID-19

## Abstract

Severe acute respiratory syndrome coronavirus-2 (SARS-CoV-2) is the etiologic agent of the coronavirus disease 2019 (COVID-19), which caused one of the pandemics with the highest mortalities with millions of deaths and hundreds of millions of cases to date. Due to its potential for airborne transmission, many studies have focused on SARS-CoV-2 primarily as a respiratory disease. However, the spread of SARS-CoV-2 to non-respiratory organs has been experimentally demonstrated and clinically observed. During autopsy studies, histopathological lesions, and disruption of the blood-testes barrier (BTB) have been observed in the male reproductive tract. Here, we review findings from both autopsy cases and animal models that demonstrate testicular disease due to COVID-19 and present an overview of the pathological alterations that occur in the testes resulting from SARS-CoV-2 infection and explore its potential mechanisms.

## 1. Introduction

Severe acute respiratory syndrome coronavirus 2 (SARS-CoV-2), the etiologic agent of coronavirus disease 2019 (COVID-19), is an enveloped positive-sense, single-stranded RNA virus that causes disease primarily affecting the respiratory tract (1–4). SARS-CoV-2 replicates in the lungs causing high levels of inflammation leading to severe damage and pathological lesions (5, 6). Widespread viral replication in both respiratory and non-respiratory tissues have been detected in multiple autopsy studies in SARS-CoV-2 patients with severe disease and can lead to extrapulmonary complications (7–10). Viral antigens in immune cell populations such as neutrophils, macrophages, B cells, T cells, and NK cells were detected in postmortem lung samples infected with SARS-CoV-2, suggesting that SARS-CoV-2 can potentially have a broad range of target cells (9, 11). Growing evidence shows SARS-CoV-2 infection in various additional tissues and organs, including the heart, brain, gastrointestinal tract, kidneys, liver, and the male reproductive tract (MRT) (12–14). Autopsies of severe COVID-19 patients contained viral RNA in multiple organs such as lungs, kidneys, lymph nodes, spleen, and testes up to 76 days, and in the brain, heart, and lungs as late as 230 days following the first signs of symptoms (7). Inflammatory cell infiltration was present in perivascular regions and the testes showed a broad spectrum of damage and reduction in spermatogenic cells (10, 15). These findings demonstrate SARS-CoV-2 is not limited to only the respiratory system but can cause systemic infections.

SARS-CoV-2 infection of the MRT could have short and long-term implications. The receptor angiotensin-converting enzyme 2 (ACE2), in addition to serine protease, transmembrane protease serine 2 (TMPRSS2), can promote SARS-CoV-2 entry through cleavage of its spike glycoprotein, (S) protein, which allows for fusion of the cell membrane (16, 17). Furthermore, the ability of SARS-CoV-2 to infect human cells through ACE2-independent receptors has been previously shown and constitutes an alternative pathway for viral entry and infection (18–20). ACE2 is primarily expressed in the cell lining of the alveoli as well as the male reproductive tract on the spermatogonia, Sertoli cells in seminiferous tubules, and Leydig cells (16, 21). This could account, in part, for why men are more impacted than females, with higher rates of mortality and greater severity of disease (22, 23), with results showing the male/female ratio for COVID-19 cases and deaths are 1.27 and 1.35, respectively (15). Studies indicate SARS-CoV-2 attachment to ACE2 facilitates the infection in the male reproductive tract (14, 24–26).

### 1.1. Comorbidities lead to possible consequences for testicular disease

Comorbidities impact COVID-19 disease severity, potentially by altering the immune response or eliciting inflammation, which can influence the male reproductive health (16, 27). Pre-existing diseases such as diabetes, obesity, hypertension, and cardiovascular disease can increase SARS-CoV-2 infection, leading to a higher risk of severe illness and histopathological damage (7, 28, 29). Among men who had confirmed COVID-19 infection, a higher proportion of individuals had pre-existing conditions such as diabetes mellitus, hyperlipidemia, hypertension, and hypogonadism (30). Additionally, diabetes and obesity are associated with lowered testosterone levels, testicular dysfunction, and inflammation, which can compromise the MRT (31). Studies have found diabetic patients with severe COVID-19 are more susceptible in developing orchiepididymitis; of these cases, testicular pain leading to necrosis in the seminiferous tubule and significant calcification in the testicular artery was found during diagnosis (32), which may suggest that COVID-19 could trigger systemic vasculitis (33). Comorbidities with SARS-CoV-2 infection may augment inflammation and render individuals more vulnerable to testicular damage (15). It is still unclear whether the testicular pathology observed in individuals with COVID-19 could be the result of a combination of the disease with pre-existing diseases.

### 1.2. Testicular infection by SARS-CoV-2

The testes are classically considered an immune-privileged organ, and the blood-testes barrier (BTB) plays an essential role in disease prevention. The testes have an immunosuppressive environment regulated by resident cells like Sertoli cells, Leydig cells, testicular macrophages, spermatogonial stem cells, and peritubular myoid cells (34). The BTB is formed by basal tight junctions between neighboring Sertoli cells within seminiferous tubules and prevents antigens and antibodies from disrupting spermatogenesis by infection or immune activation (34). Sertoli cells plays a role in supporting the production of sperm, forming a protective barrier and actively participates in the innate immune response against viral infections through the secretion of cytokines and interferons (35). Macrophages in the testes are the most diverse and abundant immune cells and have a crucial role in maintaining testicular health and function (36). Due to local immune system activity, which may result in deleterious effects in this immune-privileged organ (37), leukocyte infiltration combined with inflammatory cytokines can have detrimental effects on the function of Leydig cells, which are outside the BTB, leading to impaired testosterone production as well as damage to the BTB (38, 39). Infection with SARS-CoV-2 can be associated with a decrease in testosterone levels, testicular pain, erectile dysfunction, and histopathological lesions in severe cases (23, 30, 38–40).

SARS-CoV-2 is not unique in its ability to infect the MRT. Several other viruses, including mumps, Zika, Marburg, and Ebola, are known to infect the testes, disrupt the BTB, and are associated with cell infiltrates in the MRT (41–44), which can lead to testicular damage and affect fertility (34). However, ACE2 is not utilized by these viruses as a viral receptor, suggesting potentially several mechanisms for testicular infection which are virus dependent. ACE2 is abundantly expressed in cells of the human testes, such as spermatogonia, Leydig, and Sertoli cells (45). ACE2-positive cells exhibited increased levels of RNA transcripts associated with SARS-CoV-2 viral replication and transmission (21), thereby posing a risk to male reproductive function (29). Although the interaction between ACE2 and SARS-CoV-2 has been corroborated, a study on autopsies of COVID-19 patients showed weak immunostaining intensity of the ACE2 protein in spermatogonia, Leydig cells, Sertoli cells, and particularly in the seminiferous tubules (40). Further investigations through RT-qPCR showed that patients with more severe symptoms displayed diminished ACE2 expression, which could be due to the direct influence of SARS-CoV-2 on testicular cells, leading to the deregulation of ACE2 (40). Similarly, another study using a combination of RT-qPCR, immunohistochemistry, and transmission electron microscopy (TEM) detected the presence of SARS-CoV-2 in various organs, including the testes (46). Alternatively, samples that tested positive for SARS-CoV-2 were analyzed through immunofluorescence revealed that ACE2 receptors were inversely proportional to the level of spermatogenesis observed upon pathological examination (46). Although these two studies offer divergent results, it is crucial to acknowledge that a restricted sample size was utilized and that timing of fixation and collection may impact the variability of the results (26, 40, 45, 46). Further research endeavors are important to establish definitive and conclusive data about ACE2 in the MRT during SARS-CoV-2 infection.

Studies also examined pathological changes in testicular tissue, alterations in the testicular tunica propria, signs of orchitis, and spermatogenesis damages in patients who died or recovered from SARS-CoV-2 (15, 40, 46, 47). Achua et al. showed that three of the six biopsies had impairments of spermatogenesis. The basement membrane of the seminiferous tubules displayed thickening and hyalinization and a significant amount of lymphocyte infiltration, consistent with an autopsy study from Duarte-Neto et al. (15). These data show a greater risk of impaired spermatogenesis in men with higher levels of ACE2 receptors in the testes. Individuals infected with SARS-CoV-2 can potentially exhibit damage to seminiferous tubules, reduction of Leydig cells, reduced spermatogenesis, and lymphocytic inflammation (15, 47). In a small sample size of COVID-19 patients, the testicular tunica propria was found to be enlarged and showed a greater amount of peritubular myeloid cells and collagen fibers (40). Additionally, thickening was observed in areas close to mast cells and macrophages, both in the tunica propria and the basal membrane, and the rete testis area showed evidence of germ cell degeneration. Compromised tight junctions between Sertoli cells were observed in the seminiferous tubules, followed by the detachment of the seminiferous epithelium from the tunica propria (40). Additionally, semen parameters have been studied in COVID-19 patients (25, 29, 38, 39). A study by Gacci et al. found that all 11 patients with semen impairment had low sperm count after having COVID-19. They also observed that approximately 77% of the 33 patients displayed elevated levels of semen IL-8 (sIL-8), which is linked to inflammation. Among this group, 7 out of 12 patients who were not admitted to the hospital during their illness exhibited high levels of sIL-8. Furthermore, 80.8% of the patients requiring hospitalization and 100% of those in the intensive care unit demonstrated elevated levels of sIL-8. However, these findings also emphasized the correlation between the need for oxygen therapy and increased levels of sIL-8 (25). Ma et al. found that 4 out of 12 patients had low sperm count and lower sperm motility (29). Additionally, a study by Li et al. examined semen samples from COVID-19 patients and showed that out of 23 patients, 9 had low sperm counts. The average sperm counts in COVID-19 patients were significantly lower compared to men of similar age. Additionally, 14 patients had high levels of leukocyte in their semen. The percentage of COVID-19 patients with abnormal leukocytes levels was significantly higher than the control group. The study also measured proinflammatory cytokines and chemokines in the semen, including IL-6, tumor-necrosis-factor (TNF), and CCL2 (MCP-1), and COVID-19 patients had significantly higher levels than the control group. These findings highlight the potential consequences of COVID-19 on the testes.

### 1.3. Evidence for COVID-19-caused testicular damage and the role of the local immune response

The testicular immune response has cellular and humoral components that counter viral infections. Testicular inflammatory damage resulting from COVID-19 can potentially be an indirect result of the immune system's efforts to eliminate the virus. A study by Costa and colleagues revealed that COVID-19 patients had ten times higher levels of mast cells in their testes, suggesting that mast cells can have a role in testicular damage (40). Li and colleagues showed impaired spermatogenesis due to extensive germ cell destruction in severe COVID-19 patients autopsies. Histopathological analysis revealed thinning of the seminiferous epithelium and increased apoptotic cells and spermatogenic epithelium shedding in the seminiferous tubules (38, 39). Leukocyte infiltration can have detrimental effects on Leydig cells, leading to reduced testosterone production, damage to the blood-testis barrier, and destruction of the seminiferous epithelium (38, 39). Individuals with severe COVID-19 exhibit elevated plasma concentrations of cytokines, including interleukin (IL)-2, IL-6, IL-7, IL-10, TNF, and monocyte chemoattractant protein-1 (13). These effects, combined with inflammatory cytokines, may trigger an autoimmune response, resulting in the accumulation of IgG deposits within the tubules (38, 39). Previous research has also noted the presence of IgG deposits in various areas of the testes during experimental autoimmune orchitis (38, 39). Cytokines such as IL-6 and TNF-α can compromise the integrity of the BTB (48) and lead to orchitis and male infertility (49). Orchitis can potentially hinder the immune balance within the testes and impair the seminiferous epithelium and spermatogonia stem cells (49). A study by Basolo et al. detected SARS-CoV-2 RNA in approximately one-third of infected patient testes. CD8^+^ *T* cell infiltrates were observed within the seminiferous tubules, and clusters of CD68^+^ were present in the extra tubular space, with a notable absence of CD20^+^ and CD57^+^ cells (26). Although viral loads in the testes were low in most cases, this study demonstrated a higher occurrence of inflammatory cell infiltrates in SARS-CoV-2 infected testes compared to control subjects.

Hypogonadism and decreased testosterone levels have been found in individuals infected with SARS-CoV-2 (24). This may be linked to a disruption in the function of Leydig cells (50), which produce testosterone in response to luteinizing hormones (LH), contributing to spermatogenic differentiation (51). Additionally, androgen receptor (AR)-positive cells in the seminiferous tubules were higher in SARS-CoV-2 positive cases compared to the control group. In contrast, the percentage of follicle-stimulating hormone (FSH) receptor cells was reduced (26). Notably, the expression of luteinizing hormone/choriogonadotropin receptor (LHCGR) was significantly elevated in the testes of SARS-CoV-2 positive subjects compared to SARS-CoV-2 negative individuals and uninfected controls (26). The increased expression of LHCGR in viral positive testes may be a feedback response due to decreased levels of LH circulation in the body (26). Hormone dysfunctions may cause impaired production of testosterone and male infertility. In an *in vitro* experiment, Vero cells were exposed to testicular homogenates samples from patients who had died soon or long after the onset of COVID-19 symptoms (40). SARS-CoV-2 replication within the Vero cells and viral particles were detected in all samples. IF and TEM revealed infected monocytes/macrophages surrounding blood vessels and migrating towards testicular tissue, possibly leading to the spread of SARS-CoV-2 to testicular cells, with further detection of monocytes/macrophages inside the seminiferous tubules as another potential route of viral spread (40). The data suggest that the inflammatory damage observed in the testes may be an indirect consequence of the immune system's concerted actions to eliminate the viral presence.

### 1.4. Animal models to study SARS-CoV-2 infections of the testes and associated pathology

Analyzing viral testicular infections from patients can be challenging. Sample sizes of SARS-CoV-2 cases are frequently limited and often consist primarily of specimens from individuals with severe or lethal infections, which may not accurately represent mild to moderate cases. Additionally, the preservation methods and storage duration used in autopsies and tissue collections can impact the integrity of the specimens, affecting the results' validity. Animal models allow an evaluation of infection mechanism, and a means to test the efficacy and safety of novel therapies and vaccines. Mice and hamsters have been used as models to study SARS-CoV-2 infection of the testes and associated pathology. Hamsters are commonly used to study airborne viruses due to their pulmonary characteristics resembling those in humans; however, this model often shows milder clinical symptoms than humans (52).

Wild-type mice do not exhibit apparent pathological symptoms when infected with SARS-CoV-2 due to inefficient binding interactions between the viral spike protein and the mouse orthologue of the human ACE2 receptor (53). The use of a mouse-adapted SARS-CoV-2 (SARS-CoV-2 MA) has demonstrated disease pathogenesis and replication of SARS-CoV-2 in the upper and lower respiratory tract of mice (53). Further, some human ACE2 (hACE) transgenic mouse models have been used to study human-like COVID19 (54). Chen and colleagues show expression of hACE2 gene is regulated by mouse ACE2 (13). VSV-based SARS-CoV-2 pseudovirus (rVSV-SARS-CoV-2) testicular inoculation led to alteration and damage of the seminiferous tubules, and viral spread to the interstitial cells and eventual detection inside the seminiferous tubules, leading to the loss of germ cells and seminiferous tubule damage, thus indicating that the testis may be highly susceptible to SARS-CoV-2 infection (13).

A previous study from our group used six-to eight-week-old male golden Syrian hamsters to model SARS-CoV-2 infection (52). Testicular tissue infected *ex vivo* showed an increase in viral titers in the culture supernatant and immunofluorescence detected dsRNA, which forms during viral replication. The hamsters were infected intranasally with a target dose of 1 × 10^5^ plaque forming units (PFU) of the original SARS-CoV-2, USA/WA1/2020 strain. Although viral detection was not present in plaque assays, SARS-CoV-2 RNA was detected in testicular tissue in all samples 2- and 4-days post-infection (dpi) by RT-qPCR. Moreover, due to the low level of infectivity, notable pathological changes were not shown in the hamster testes, and was insufficient to result in direct adverse effects or elicit a substantial immunological response (52). Furthermore, age-dependent differences have been shown in younger hamsters eliciting a stronger immune response when compared to older hamsters which may also be a contributing factor to the observed outcome (55). A separate study by Li et al. also investigated pathological and immune damage in six-to eight-week-old male hamster testes. The hamsters were challenged by SARS-CoV-2 and its variants, Delta (B.1.617.2) and Omicron (B1.1.529), through intranasal with 10^3^ PFU/ml or testicular inoculation with 10^5^ PFU/ml and analyzed at 1 dpi to 120 dpi (48). Intranasal exposure resulted in a significant reduction of sperm count at and decreased levels of testosterone at 4 dpi, and a gradual reduction in testicular size and weight at 120 dpi (48). An increase in virus inoculation resulted in severe histopathological damage and inflammation of the testes, leading to the deterioration of the seminiferous tubules and the absence of Sertoli and Leydig cells (48). In this study, infections with SARS-CoV-2 in Syrian hamsters demonstrated acute testicular damage, chronic testicular atrophy, and decreased hormones (48). These findings support the use of the Syrian hamster model as a valid tool for understanding the pathogenesis of SARS-CoV-2 and testing vaccines or antiviral drugs (56). Animal models are a crucial tool for studying the effects on testicular cells as they model organ systems more effectively than *in vitro* models (45). Overall, further advancements in existing models, as well as the development of additional models could improve the scope and depth of ongoing studies.

## 2. Conclusion

While the immune response is crucial in defending the body from pathogens, severe COVID-19 may trigger testicular inflammation and histopathological damage. Since the outbreak of the COVID-19 pandemic, numerous research studies have been studied to gain a better understanding of the disease's progression, identify effective treatments, and develop strategies to mitigate transmission, including in the MGT. Studies suggest that severe cases of COVID-19 can lead to testicular damage, potentially due to direct infection of the testicular cells by the SARS-CoV-2 virus or *via* infected immune cells, and subsequent immune overactivation. Additionally, the immunological response elicited by the infection with SARS-CoV-2 can result in testicular dysfunction, thereby damaging reproductive health. These effects may be more severe in individuals with co-morbidities that increase the resting inflammatory state. Animal models are currently being improved to help investigate pathology and to evaluate the efficacy of therapies to treat COVID-19. Further research is crucial for developing effective treatments and preventative measures to reduce the negative impact of SARS-CoV-2 infection on reproductive health.

## References

[1] Cao C Cai Z Xiao X Rao J Chen J Hu N The architecture of the SARS-CoV-2 RNA genome inside virion. Nat Commun. (2021) 12(1):3917. 10.1038/s41467-021-22785-x 34168138 PMC8225788

[2] Lu R Zhao X Li J Niu P Yang B Wu H Genomic characterisation and epidemiology of 2019 novel coronavirus: implications for virus origins and receptor binding. Lancet. (2020) 395(10224):565–74. 10.1016/S0140-6736(20)30251-8 32007145 PMC7159086

[3] Zhu N Zhang D Wang W Li X Yang B Song J A novel coronavirus from patients with pneumonia in China, 2019. N Engl J Med. (2020) 382(8):727–33. 10.1056/NEJMoa2001017 31978945 PMC7092803

[4] Zhou P Yang X-L Wang X-G Hu B Zhang L Zhang W A pneumonia outbreak associated with a new coronavirus of probable bat origin. Nature. (2020) 579(7798):270–73. 10.1038/s41586-020-2012-7 32015507 PMC7095418

[5] Zhang Y Liu Y Gong H Wu L. “Quantitative lung lesion features and temporal changes on chest CT in patients with common and severe SARS-CoV-2 pneumonia.” edited by raffaele Serra. PLOS ONE. (2020) 15(7): e0236858. 10.1371/journal.pone.0236858 32706819 PMC7380626

[6] Pan F Ye T Sun P Gui S Liang B Li L Time course of lung changes at chest CT during recovery from coronavirus disease 2019 (COVID-19). Radiology. (2020) 295(3):715–21. 10.1148/radiol.2020200370 32053470 PMC7233367

[7] Stein SR Ramelli SC Grazioli A Chung J-Y Singh M Yinda CK SARS-CoV-2 infection and persistence in the human body and brain at autopsy. Nature. (2022) 612(7941):758–63. 10.1038/s41586-022-05542-y 36517603 PMC9749650

[8] Elrobaa IH New KJ. COVID-19: pulmonary and extra pulmonary manifestations. Front Public Health. (2021) 9(September):711616. 10.3389/fpubh.2021.711616 34650947 PMC8505777

[9] Ren X Wen W Fan X Hou W Su B Cai P COVID-19 Immune features revealed by a large-scale single-cell transcriptome atlas. Cell. (2021) 184(7):1895–1913.e19. 10.1016/j.cell.2021.01.053 33657410 PMC7857060

[10] Bian X-W. Autopsy of COVID-19 patients in China. Natl Sci Rev. (2020) 7(9):1414–18. 10.1093/nsr/nwaa123 34192086 PMC7313767

[11] Shen X-R Geng R Li Q Chen Y Li S-F Wang Q ACE2-Independent Infection of T lymphocytes by SARS-CoV-2. Signal Transduction and Targeted Therapy. (2022) 7(1):1–11. 10.1038/s41392-022-00919-x 35277473 PMC8914143

[12] Gupta A Madhavan MV Sehgal K Nair N Mahajan S Sehrawat TS Extrapulmonary manifestations of COVID-19. Nat Med. (2020) 26(7):1017–32. 10.1038/s41591-020-0968-3 32651579 PMC11972613

[13] Chen M Li S Liu S Zhang Y Cui X Lv L “Infection of SARS-CoV-2 causes severe pathological changes in mouse testis.” journal of genetics and genomics. December. (2022) 50:99–107. 10.1016/j.jgg.2022.11.011 PMC9724560 36494057

[14] Qi J Zhou Y Hua J Zhang L Bian J Liu B The ScRNA-seq expression profiling of the receptor ACE2 and the cellular protease TMPRSS2 reveals human organs susceptible to SARS-CoV-2 infection. Int J Environ Res Public Health. (2021) 18(1):284. 10.3390/ijerph18010284 33401657 PMC7794913

[15] Duarte-Neto AN Teixeira TA Caldini EG Kanamura CT Gomes-Gouvêa MS Santos A.B. D Testicular pathology in fatal COVID-19: a descriptive autopsy study. Andrology. (2022) 10(1):13–23. 10.1111/andr.13073 34196475 PMC8444746

[16] Aleksova A Gagno G Sinagra G Beltrami AP Janjusevic M Ippolito G Effects of SARS-CoV-2 on cardiovascular system: the dual role of angiotensin-converting enzyme 2 (ACE2) as the virus receptor and homeostasis regulator-review. Int J Mol Sci. (2021) 22(9):4526. 10.3390/ijms22094526 33926110 PMC8123609

[17] Hoffmann M Kleine-Weber H Schroeder S Krüger N Herrler T Erichsen S SARS-CoV-2 cell entry Depends on ACE2 and TMPRSS2 and is blocked by a clinically proven protease inhibitor. Cell. (2020) 181(2):271–280.e8. 10.1016/j.cell.2020.02.052 32142651 PMC7102627

[18] Lim S Zhang M Chang TL. ACE2-Independent Alternative receptors for SARS-CoV-2. Viruses. (2022) 14(11):2535. 10.3390/v14112535 36423144 PMC9692829

[19] Hoffmann M Sidarovich A Arora P Krüger N Nehlmeier I Kempf A Evidence for an ACE2-independent entry pathway that can protect from neutralization by an antibody used for COVID-19 therapy. MBio. (2022) 13(3):e00364–22. 10.1128/mbio.00364-22 35467423 PMC9239067

[20] Liu J Lu F Chen Y Plow E Qin J. Integrin mediates cell entry of the SARS-CoV-2 virus independent of cellular receptor ACE2. J Biol Chem. (2022) 298:3. 10.1016/j.jbc.2022.101710 PMC8828381 35150743

[21] Wang Z Xu X. ScRNA-Seq profiling of human testes reveals the presence of the ACE2 receptor, A target for SARS-CoV-2 infection in spermatogonia, leydig and sertoli cells. Cells. (2020) 9(4):920. 10.3390/cells9040920 32283711 PMC7226809

[22] Chakravarty D Nair SS Hammouda N Ratnani P Gharib Y Wagaskar V Sex differences in SARS-CoV-2 infection rates and the potential link to prostate cancer. Communications Biol. (2020) 3(1):1–12. 10.1038/s42003-020-1088-9 PMC7343823 32641750

[23] Meng Y Wu P Lu W Liu K Ma K Huang L Sex-Specific clinical characteristics and prognosis of coronavirus disease-19 infection in Wuhan, China: a retrospective study of 168 severe patients. PLoS Pathog. (2020) 16(4):e1008520–e1008520. 10.1371/journal.ppat.1008520 32343745 PMC7209966

[24] Dutta S Sengupta P. SARS-CoV-2 and male infertility: possible multifaceted pathology. Reprod Sci. (2020) 28(1):23–6. 10.1007/s43032-020-00261-z 32651900 PMC7351544

[25] Gacci M Coppi M Baldi E Sebastianelli A Zaccaro C Morselli S Semen impairment and occurrence of SARS-CoV-2 virus in semen after recovery from COVID-19. Human Reprod (Oxford, England). (2021):deab026. 10.1093/humrep/deab026 PMC7953947 33522572

[26] Basolo A Poma AM Macerola E Bonuccelli D Proietti A Salvetti A Autopsy study of testicles in COVID-19: upregulation of immune-related genes and downregulation of testis-specific genes. J Clin Endocrinol Metab. (2022):dgac608. 10.1210/clinem/dgac608 PMC9620766 36260523

[27] Deng Y Liu W Liu K Fang Y-Y Shang J Zhou L Clinical characteristics of fatal and recovered cases of coronavirus disease 2019 in Wuhan, China: a retrospective study. Chin Med J. (2020) 133(11):1261–67. 10.1097/CM9.0000000000000824 32209890 PMC7289311

[28] Barron E Bakhai C Kar P Weaver A Bradley D Ismail H Associations of type 1 and type 2 diabetes with COVID-19-related mortality in England: a whole-population study. Lancet Diabetes Endocrinol. (2020) 8(10):813–22. 10.1016/S2213-8587(20)30272-2 32798472 PMC7426088

[29] Ma Y Lu D Bao L Qu Y Liu J Qi X SARS-CoV-2 infection aggravates chronic comorbidities of cardiovascular diseases and diabetes in mice. Animal Models and Experimental Med. (2021) 4(1):2–15. 10.1002/ame2.12155 PMC7954823 33738432

[30] Hebert KJ Matta R Horns JJ Paudel N Das R McCormick BJ “Prior COVID-19 infection associated with increased risk of newly diagnosed erectile dysfunction. Int J Impot Res. (2023) 10:1–5. 10.1038/s41443-023-00687-4 PMC10015534 36922696

[31] Jiang Q ThomasLinn KD Shi L. Diabetes as a potential compounding factor in COVID-19-mediated male subfertility. Cell Biosci. (2022) 12(1):35. 10.1186/s13578-022-00766-x 35307018 PMC8934536

[32] Zhang TR Thorogood SL Miyauchi J Del Pizzo J Schlegel PN. Acute testicular infarction in the setting of SARS-CoV-2 infection and diabetic vasculopathy. Urol Case Rep. (2023) 47(February):102342. 10.1016/j.eucr.2023.102342 36748071 PMC9891301

[33] Salazar A Gonzalez A Murray NP Castro C. Atypical presentation of COVID-19: chronic bilateral testicular pain with lower extremity peripheral polyneuropathy, case report. Urol Case Rep. (2021) 40(November):101932. 10.1016/j.eucr.2021.101932 34778003 PMC8576329

[34] Kaur G Wright K Verma S Haynes A Dufour JM. The good, the bad and the ugly of testicular immune regulation: a delicate balance between immune function and immune privilege. Adv Exp Med Biol. (2021) 1288:21–47. 10.1007/978-3-030-77779-1_2 34453730

[35] Sun B Qi N Shang T Wu H Deng T Han D. Sertoli cell-initiated testicular innate immune response through toll-like receptor-3 activation is negatively regulated by Tyro3, axl, and mer receptors. Endocrinology. (2010) 151(6):2886–97. 10.1210/en.2009-1498 20363878

[36] Bhushan S Theas MS Guazzone VA Jacobo P Wang M Fijak M Immune cell subtypes and their function in the testis. Front Immunol. (2020):11. 10.3389/fimmu.2020.583304 33101311 PMC7554629

[37] Ediz C Tavukcu HH Akan S Kizilkan YE Alcin A Oz K Is there any association of COVID-19 with testicular pain and epididymo-orchitis? Int J Clin Pract. (2021) 75(3):e13753. 10.1111/ijcp.13753 33063899 PMC7646040

[38] Li H Xiao X Zhang J Zafar MI Wu C Long Y Impaired spermatogenesis in COVID-19 patients. EClinicalMedicine. (2020a) 28(November):100604. 10.1016/j.eclinm.2020.100604 33134901 PMC7584442

[39] Li X Geng M Peng Y Meng L Lu S. Molecular immune pathogenesis and diagnosis of COVID-19. J Pharm Anal. (2020b) 10(2):102–8. 10.1016/j.jpha.2020.03.001 32282863 PMC7104082

[40] Costa GMJ Lacerda SMSN Figueiredo AFA Wnuk NT Brener MRG Andrade LM High SARS-CoV-2 tropism and activation of immune cells in the testes of non-vaccinated deceased COVID-19 patients. BMC Biol. (2023) 21(1):36. 10.1186/s12915-022-01497-8 36797789 PMC9933832

[41] Coffin KM Liu J Warren TK Blancett CD Kuehl KA Nichols DK Persistent marburg virus infection in the testes of nonhuman primate survivors. Cell Host Microbe. (2018) 24(3):405–416.e3. 10.1016/j.chom.2018.08.003 30173956

[42] Schindell B. G. Webb A. L. Kindrachuk J, and this link will open in a new window Link to external site. “Persistence and sexual transmission of filoviruses.” Viruses. (2018) 10(12):683. 10.3390/v10120683 30513823 PMC6316729

[43] Wu H Jiang X Gao Y Liu W Wang F Gong M Mumps virus infection disrupts blood-testis barrier through the induction of TNF-α in sertoli cells. FASEB J. (2019) 33(11):12528–40. 10.1096/fj.201901089R 31450968 PMC6902681

[44] Tsetsarkin KA Acklin JA Liu G Kenney H Teterina NL Pletnev AG Zika Virus tropism during early infection of the testicular interstitium and its role in viral pathogenesis in the testes. PLoS Pathog. (2020) 16(7):e1008601. 10.1371/journal.ppat.1008601 32614902 PMC7331987

[45] Edenfield RC Easley CA. Implications of testicular ACE2 and the renin–angiotensin system for SARS-CoV-2 on testis function. Nat Rev Urol. (2022) 19(2):116–27. 10.1038/s41585-021-00542-5 34837081 PMC8622117

[46] Achua JK Chu KY Ibrahim E Khodamoradi K Delma KS Iakymenko OA Histopathology and ultrastructural findings of fatal COVID-19 infections on testis. The World Journal of Men's Health. (2021) 39(1):65–74. 10.5534/wjmh.200170 PMC7752514 33151050

[47] Yang M Chen S Huang B Zhong J-M Su H Chen Y-J Pathological findings in the testes of COVID-19 patients: clinical implications. Eur Urol Focus. (2020) 6(5):1124–29. 10.1016/j.euf.2020.05.009 32563676 PMC7261470

[48] Li C Ye Z Zhang AJ-X Chan JF-W Song W Liu F Severe acute respiratory syndrome coronavirus 2 (SARS-CoV-2) infections by intranasal or testicular inoculation induces testicular damage preventable by vaccination in golden Syrian hamsters. Clin Infect Dis. (2022) 75:ciac142. 10.1093/cid/ciac142 35178548 PMC8903466

[49] Tian Y Zhou L-q. Evaluating the impact of COVID-19 on male reproduction. Reproduction. (2021) 161(2):R37–44. 10.1530/REP-20-0523 33434886

[50] Mäkelä J-A Koskenniemi JJ Virtanen HE Toppari J. Testis development. Endocr Rev. (2019) 40(4):857–905. 10.1210/er.2018-00140 30590466

[51] Zirkin BR Papadopoulos V. Leydig cells: formation, function, and regulation. Biol Reprod. (2018) 99(1):101–11. 10.1093/biolre/ioy059 29566165 PMC6044347

[52] Campos RK Camargos VN Azar SR Haines CA Eyzaguirre EJ Rossi SL. SARS-CoV-2 infects hamster testes. Microorganisms. (2021) 9(6):1318. 10.3390/microorganisms9061318 34204370 PMC8235703

[53] Dinnon KH Leist SR Schäfer A Edwards CE Martinez DR Montgomery SA A mouse-adapted model of SARS-CoV-2 to test COVID-19 countermeasures. Nature. (2020) 586(7830):560–66. 10.1038/s41586-020-2708-8 32854108 PMC8034761

[54] Bao L Deng W Huang B Gao H Liu J Ren L The pathogenicity of SARS-CoV-2 in HACE2 transgenic mice. Nature. (2020) 583(7818):830–33. 10.1038/s41586-020-2312-y 32380511

[55] Osterrieder N Bertzbach LD Dietert K Abdelgawad A Vladimirova D Kunec D Age-Dependent progression of SARS-CoV-2 infection in Syrian hamsters. Viruses. (2020) 12(7):779. 10.3390/v12070779 32698441 PMC7412213

[56] Imai M Iwatsuki-Horimoto K Hatta M Loeber S Halfmann PJ Nakajima N Syrian Hamsters as a small animal model for SARS-CoV-2 infection and countermeasure development. Proc Natl Acad Sci U S A. (2020) 117(28):16587–95. 10.1073/pnas.2009799117 32571934 PMC7368255

